# Genome-Wide Association Study Reveals Novel Powdery Mildew Resistance Loci in Bread Wheat

**DOI:** 10.3390/plants12223864

**Published:** 2023-11-15

**Authors:** Ramandeep Kaur, Neeraj Kumar Vasistha, Vikas Kumar Ravat, Vinod Kumar Mishra, Sandeep Sharma, Arun Kumar Joshi, Raman Dhariwal

**Affiliations:** 1 Department of Genetics-Plant Breeding and Biotechnology, Dr. Khem Sigh Gill Akal College of Agriculture, Eternal University, Baru Sahib, Sirmour 173101, India; 2 Department of Genetics and Plant Breeding, Rajiv Gandhi University, Rono Hills, Itanagar 791112, India; 3 Department of Plant Pathology, Rajiv Gandhi University, Rono Hills, Itanagar 791112, India; 4 Department of Genetics and Plant Breeding, Institute of Agricultural Sciences, Banaras Hindu University, Varanasi 221005, India; 5 Borlaug Institute for South Asia (BISA), NASC Complex, DPS Marg, New Delhi 110012, India; 6 International Maize and Wheat Improvement Center (CIMMYT) Regional Office, NASC Complex, DPS Marg, New Delhi 110012, India; 7 Agriculture and Agri-Food Canada, Lethbridge Research and Development Centre, 5403 1 Avenue South, Lethbridge, AB T1J 4B1, Canada

**Keywords:** powdery mildew, wheat, GWAS, FarmCPU, MTAs, candidate genes

## Abstract

Powdery mildew (PM), caused by the fungal pathogen *Blumeria graminis* f. sp. *tritici* (*Bgt*), significantly threatens global bread wheat production. Although the use of resistant cultivars is an effective strategy for managing PM, currently available wheat cultivars lack sufficient levels of resistance. To tackle this challenge, we conducted a comprehensive genome-wide association study (GWAS) using a diverse panel of 286 bread wheat genotypes. Over three consecutive years (2020–2021, 2021–2022, and 2022–2023), these genotypes were extensively evaluated for PM severity under field conditions following inoculation with virulent *Bgt* isolates. The panel was previously genotyped using the Illumina 90K Infinium iSelect assay to obtain genome-wide single-nucleotide polymorphism (SNP) marker coverage. By applying FarmCPU, a multilocus mixed model, we identified a total of 113 marker–trait associations (MTAs) located on chromosomes 1A, 1B, 2B, 3A, 3B, 4A, 4B, 5A, 5B, 6B, 7A, and 7B at a significance level of *p* ≤ 0.001. Notably, four novel MTAs on chromosome 6B were consistently detected in 2020–2021 and 2021–2022. Furthermore, within the confidence intervals of the identified SNPs, we identified 96 candidate genes belonging to different proteins including 12 disease resistance/host–pathogen interaction-related protein families. Among these, protein kinases, leucine-rich repeats, and zinc finger proteins were of particular interest due to their potential roles in PM resistance. These identified loci can serve as targets for breeding programs aimed at developing disease-resistant wheat cultivars.

## 1. Introduction

Bread wheat (*Triticum aestivum* L.) is a vital source of nutrients and serves as the primary staple food crop for approximately 35% of the global population [1]. In 2022, worldwide wheat consumption increased from 783 million metric tons to 792.69 million metric tons [2]. This rise in wheat consumption is necessary for global food security, but has led to extra burdens in the wheat production system, which is already challenged by the prevalence of pests and diseases such as powdery mildew (PM), a devastating wheat disease.

PM is caused by the fungal pathogen *Blumeria graminis* (DC.) E.O. Speer f. sp. *tritici* Em. Marchal (syn. *Erysiphe graminis* f. sp. *tritici*) (*Bgt*). It is the third most destructive disease of wheat, causing yield losses ranging from 13% to 34% under low infestation and 50% to 100% under severe infestation in various wheat-growing regions [3,4]. Environmental factors like temperature and relative humidity significantly influence the development and spread of this disease, contributing to epidemics [5]. In the Himalayan regions of India, PM is a significant factor contributing to reduced grain yield [6]. Breeding approaches have proven effective in developing PM-resistant wheat varieties to overcome yield losses and increase economic production. Using resistant varieties is currently considered an efficient, environmentally safe, and successful strategy to control PM and mitigate production losses.

Major effect PM resistance genes and minor- to moderate-effect PM resistance genes are generally inherited qualitatively and quantitatively, respectively, and have also been identified in wheat and integrated into wheat resistance breeding programs. These include over 100 alleles distributed over >60 major loci (*Pm1*–*Pm68*: *Pm8* is allelic to *Pm17*; *Pm18* = *Pm1c*; *Pm22* = *Pm1e*; *Pm23* = *Pm4c*, and *Pm31* = *Pm21*) and many valuable quantitative trait loci (QTLs) distributed across wheat chromosomes [7,8,9,10,11,12,13,14,15,16,17,18,19,20,21,22,23,24,25]. Whereas some PM resistance genes, such as *Pm38*, *Pm39*, and *Pm46*, are inherited quantitatively [26,27,28], there are other genes which possess many resistance alleles. For instance, *Pm3* exhibits the highest allelic diversity, with 17 variants, followed by *Pm1* and *Pm5*, each possessing 5 alleles. *Pm4* and *Pm2*, on the other hand, possess four and three alleles, respectively [11].

PM resistance genes have primarily been transferred from both domesticated and wild relatives and from other species such as rye (*Secale cereale*) to wheat [29]. However, their utilization in wheat breeding is challenged by linkage drags [15]. For instance, the *Pm8* gene, which derived from *S. cereale* and also significantly contributed to PM resistance in wheat breeding during the 1990s, is linked with secalin glycopeptide locus on 1RS chromosome segment which causes a decline in flour quality [30]. Furthermore, many of the identified resistance genes have been overcome by the pathogen in different countries, leaving only a few that are still effective against current *Bgt* isolates in the field [31,32]. Thus, the continuous identification, characterization and deployment of new resistance genes is essential to minimize economic losses.

The declining cost of genotyping and the increasing availability of diverse single-nucleotide polymorphism (SNP) fingerprinting platforms has furthered the identification and deployment of new resistance genes. Many genetic mapping methods are now available to accommodate different types of traits, mapping populations, and study objectives [33]. These methods can be categorized into three main groups based on the principle and genetic material used: (i) QTL mapping using a biparental population, (ii) association mapping or genome-wide association studies (GWAS) in a diverse set of germplasm, and (iii) genetic mapping utilizing the multiparent populations. The first two approaches, QTL mapping and GWAS, have successfully identified genes and their markers associated with disease resistance [34,35,36,37]. However, the QTL mapping approach, which relies on a segregating population generated from parents exhibiting contrasting trait performances [12], faces limitations in QTL detection power. The scarcity of recombination events and genetic variation in such populations often leads to identifying only a few QTLs. To overcome this limitation, advanced wheat populations such as ‘multiparent advanced generation inter-cross’ (MAGIC) have been introduced to enhance the resolution of traditional mapping [38]. Nevertheless, challenges arise in validating map order and analyzing recombination due to using bi-allelic markers in these populations [39]. Conversely, GWAS circumvents the need for developing populations by conducting analyses on diverse germplasm [40] and offers several advantages such as the utilization of larger sample sizes, which facilitates the exploration of a broader range of genetic variations including those with minor effects and allelic diversity [36,41]. Additionally, GWAS enables higher-resolution mapping, identifying candidate genes associated with the trait of interest [42].

Thus, the objectives of the present study were to (i) identify potentially novel loci that confer effective PM resistance at the adult plant stage utilizing a panel of 286 wheat accessions known as the wheat association mapping initiative (WAMI) panel created by CIMMYT, Mexico, (ii) compare identified loci with the previously reported genes/QTLs, and (iii) identify potential candidate genes associated with PM resistance using comparative analyses. Leveraging landmark resources such as the high-density Illumina 90K Infinium iSelect assay [43] and the Ensemble plants database [44], we aim to address the aforementioned objectives and shed light on the genetic basis of PM resistance in wheat using a GWAS study. 

## 2. Results

### 2.1. Phenotypic Analysis

**Figure 1:**
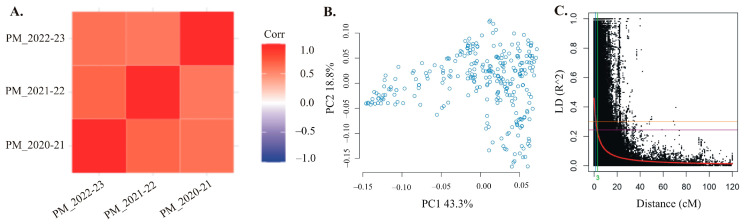
Correlation coefficient, population structure and linkage disequilibrium (LD). (**A**). A graphical representation of correlation coefficient among three environmental years for powdery mildew disease severity. (**B**). Scatterplots showing the results of principal component analysis (PCA) conducted on genotypic data obtained from the WAMI panel containing 286 wheat accessions. The figure highlights the population structure of the WAMI panel, as revealed by the first two principal components (PC1 and PC2). (**C**). A scatter plot for r^2^ values of pairwise markers showing genome-wide linkage disequilibrium decay in 286 genotypes of the WAMI panel assessed using the Hill and Weir formula [45]. The red curve line is the smoothing spline regression model fitted to LD decay. The horizontal yellow line is the standard critical r^2^ value of the genome (r^2^ = 0.3), and the vertical blue line is the genetic distance (1.5 centimorgan, cM) at the intersect between the standard critical and the LD decay curve. The vertical green line is the genetic distance (3.0 cM) at which the LD half-decay (r^2^ = 0.23, the horizontal purple line) intersects with the LD decay curve.

The phenotypic analysis conducted in specific environments showed significant variation for disease severity in the WAMI panel. Table 1 provides a summary of the phenotypic variation for PM disease severity. The broad-sense heritability for PM disease severity ranged from 65% to 89% (Table 1). The analysis of variance indicated that both genotype and environment as well as genotype–environment interactions had highly significant (*p* ≤ 0.001) estimated variance components for disease response to PM (Table 2). However, despite environmental variation, significant positive correlations (>0.6 for each environment) were observed for the disease severity among the three environments (Figure 1A).

### 2.2. Population Structure Analysis

Principal components analysis revealed largely even distribution for the allele frequencies within the WAMI panel (Figure 1B). PC1 accounted for 43.3% of the total variance, while PC2 explained 18.8%. These results suggest that the combination of PC1 and PC2 captures a significant portion of the underlying genetic variation within the panel, allowing for identifying and differentiating the subgroups present in the WAMI population.

### 2.3. Genome-Wide Marker–Trait Association (MTA) Analysis

A scatter plot for r^2^ values of pairwise markers showing genome-wide linkage disequilibrium (LD) decay for 286 genotypes of the WAMI panel assessed using the Hill and Weir formula [45]. LD analysis was performed on data generated from TASSEL using a 100-SNP sliding window. The average genome-wide r^2^ was 0.11, and LD decay began at r^2^ = 0.46 and reached half-decay at r^2^ = 0.23 (Figure 1C). The LD decay curve intersected with the half-decay and standard critical (r^2^ = 0.3) lines at 3.0 and 1.5 centimorgans (cM), respectively. This defines 1.5 cM as the genome-wide critical distance to detect linkage as described earlier [46].

**Figure 2:**
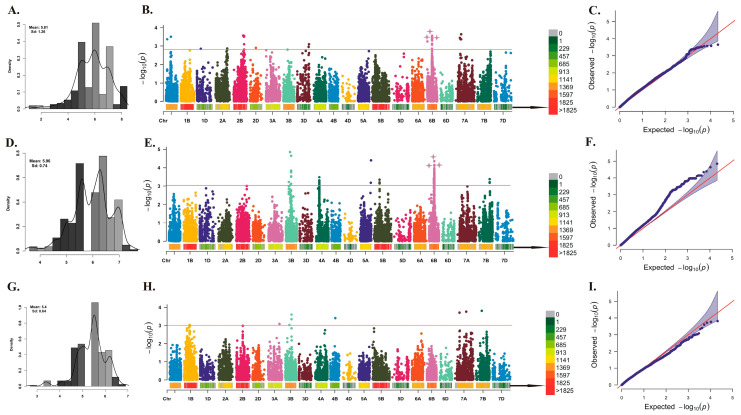
PM disease severity distribution, marker–trait associations and quantile–quantile (Q–Q) plot distributions. Histograms on the left (**A**,**D**,**G**) display the frequency distribution of BLUP estimated for PM disease severity for three consecutive years, 2020–2021, 2021–2022, and 2022–2023. Manhattan plots in the middle (**B**,**E**,**H**) illustrate the results of genome-wide association scans for PM resistance using BLUP of the 286 lines of the WAMI panel across 2020–2021, 2021–2022, and 2022–2023, respectively. The horizontal orange line represents FDR-adjusted *p* < 0.001. Associated SNP markers (above threshold) that are common among years are highlighted by star signs on the Manhattan plots. Colored bars under the Manhattan plot represent the density of markers. The Q–Q plots of *p*-values on the right (**C**,**F**,**I**) compare the observed −lg (P) for PM resistance to *Bgt* isolates in FarmCPU analyses to the expected values of −lg (P) under a uniform distribution.

The comprehensive genome-wide MTA analysis encompassed three distinct models: the general linear model (GLM), mixed linear model (MLM), and the fixed and random model circulating probability unification (FarmCPU); however, the final GWAS analysis exclusively incorporated the best-fitting FarmCPU model. Over the course of the three-year investigation, a total of 19, 85, and 9 MTAs were successfully identified in each respective year (Figure 2 and Table 3). Figure 2 displays the frequency distribution of the best linear unbiased prediction (BLUP) values for PM disease severity for three consecutive years namely, 2020–2021, 2021–2022, and 2022–2023, the Manhattan plot illustrating the results of genome-wide association scans, and the quantile–quantile (Q–Q) plot of *p*-values, comparing the observed −lg (P) for PM resistance to *Bgt* isolates in FarmCPU method.

In the initial year (2020–2021), the distribution of MTAs spanned multiple chromosomes, with notable occurrences on the chromosome 1A (2 MTAs), 2B (4 MTAs), 6B (9 MTAs), and 7A (4 MTAs). Notably, a striking concentration of 9 MTAs emerged at a specific genetic position (59.0–60.0 cM) on chromosome 6B. This intriguing observation suggests the presence of a potential genomic “hotspot” linked to PM resistance, signifying the collective impact of multiple genetic variants.

Transitioning to the subsequent year (2021–2022), the landscape of MTAs extended across various chromosomes: chromosome 2B (1 MTA), 3B (9 MTAs), 4A (19 MTAs), 5A (1 MTA), 5B (2 MTAs), 6B (48 MTAs), and 7B (3 MTAs). Noteworthy is the aggregation of MTAs at 48.0 cM on chromosome 4A, underscoring another genomic hotspot exerting influence on PM resistance. The 6B chromosome also exhibited remarkable association richness, hosting 49 MTAs. Within this cluster, 8 MTAs were anchored at 59.0 cM, 77 MTAs at 64.0 cM, and 3 MTAs at 65.0 cM, further reinforcing the pivotal role of the 6B chromosome region in contributing to the observed phenotypic variance for PM resistance.

Concluding this study in the final year (2022–2023), a reduced yet significant number of MTAs were identified, totaling 9 associations distributed across chromosomes 1B (1 MTA), 3A (1 MTA), 3B (3 associations), 7A (2 MTAs), and 7B (1 MTA).

Interestingly, four common MTAs were consistently observed in crop seasons 2020–2021 and 2021–2022 located on chromosome 6B (Table 3). Notably, certain chromosomal regions, such as 2B (132.0–144.0 cM), 3B (57.0–71.0 cM), 6B (59.0–65.0 cM), and 7A (57.0–74.0 cM), also exhibited repeated associations with PM resistance, indicating their crucial role in the genetic variation in PM resistance. 

### 2.4. Candidate Genes

In our study, we investigated genomic regions to find candidate genes within a specific window of 200 kilobases (kb) surrounding each MTA for their association with PM resistance. This window consisted of 100 kb on each side of the MTA. We discovered a total of 94 unique candidate genes (89 encoding known protein domains) in 51 genomic intervals for 65 MTAs (Table S1), while no candidate gene were spotted in defined genomic regions of the remaining 48 MTAs. 

Further analysis of these 94 candidate genes involved screening them for the presence of genes that are already known to play a role in various pathways related to the interactions between pathogens and their host organisms. The results of this screening, as presented in Table S2 revealed that 30 candidate genes encoded a diverse range of protein domains relevant to plant defense and the interactions between pathogens and hosts. The identified proteins encompassed a wide array of functional domains and families. They included the following: (i) ABC transporter-like, ATP-binding domain; (ii) Ankyrin repeat; (iii) aspartic peptidase A1 family; (iv) peroxidase; (v) cytochrome P450; (vi) disease resistance protein (NB-LRR); (vii) glutathione S-transferase; (viii) P-loop-containing nucleoside triphosphate hydrolase; (ix) kinase-like domain superfamily (including serine-threonine/tyrosine/cysteine-protein kinase); (x) wall-associated receptor kinase, galacturonan-binding domain; (xi) zinc finger proteins; (xii) F-box superfamily. These candidate genes and their corresponding proteins are directly or indirectly involved in the response of the host organism to pathogen attacks. 

## 3. Discussion

To uncover novel PM resistance genes/MTAs, a series of field experiments were conducted over three years, 2020–2021, 2021–2022, and 2022–2023, utilizing the diverse WAMI panel of common wheat. The analysis of PM disease severity unveiled a wide spectrum of phenotypic variations, displaying normal distributions in two of the three environments (2020–2021 and 2022–2023), and a near binomial distribution in the remaining environment (2021–2022) (Figure 2). This distribution pattern suggests the involvement of major and multiple quantitative trait loci (QTLs)/genes that contribute to PM resistance. As indicated by the phenotypic analysis, GWAS analysis also identified a total of 113 MTAs associated with adult plant resistance against PM (Table 3). This can be explained by the extensive diversity present for PM resistance within the WAMI panel [63]. The presence of multiple PM resistance QTLs/MTAs in wheat germplasm sourced from diverse countries has also been reported previously [16], underlining the intricate and diverse genetic foundation of PM resistance. These findings highlight the complexity and diversity of the genetic basis underlying PM resistance and reinforce the importance of the WAMI panel in dissecting PM resistance and emphasizing the need for further investigations to unravel the specific loci and PM resistance mechanisms involved in wheat.

### 3.1. Population Structure Analysis

Population structure is a crucial consideration in GWAS to mitigate false-positive as-sociations [64]. Previously, the WAMI genotype panel has been analyzed using the same marker set employing STRUCTURE software v2.3.4 [65]. Thus, in this study, to avoid redundancy, we opted for PCA analysis, which revealed that the allele frequencies were largely evenly distributed, indicating a lack of distinct subpopulations due to the absence of strong correlations among the accessions within the panel. In contrast, Singh et al. [65], employed a model-based analysis using STRUCTURE software v2.3.4, identifying six distinct subpopulations with varying genetic purity and admixture. These results could be attributed to the composition of the panel, which includes many synthetically derived lines [64], possibly corresponding to elite parents. Conversely, the divergence between these two sets of results underscores the importance of the methodologies employed. Our PCA analysis provides an overarching view of genetic diversity without highlighting subpopulations, while previous model-based analysis dissects the panel into distinct subgroups, revealing the intricate population structure. While this panel has been successfully used for GWAS for spot blotch resistance previously, we found it suitable for this study on PM resistance.

### 3.2. Investigating the Concordance among Significant MTAs and Previously Mapped QTL/Pm Resistance Genes

This study identified a total of 113 MTAs (19, 85 and 9 MTAs in 2020–2021, 2021–2022 and 2022–2023, respectively) with some notable findings such as common associated markers over the years (Table 3). We observed that the highest number (85) of MTAs were detected in the second year (2021–2022), possibly because the presence of multiple markers at the same position in linkage disequilibrium [66]. As indicated by the slightly higher disease pressure in the second year (Figure 2), the more favorable environmental conditions might have also contributed to this outcome.

Upon comparing the position of markers associated with significant MTAs identified in this study with the previously reported MTAs/QTLs and *Pm* resistance genes, it was observed that several MTAs were located to the positions where *Pm* resistance genes/QTLs had been reported previously while others represent novel loci. For instance, on chromosome 1A, we identified an MTA at the 27.0 cM position during the 2020–2021 season. This MTA shares a location with previously known *Pm* resistance loci, including resistance genes like *Pm3* [47] and *Pm223389* [48], as well as QTLs such as *QPm.mgb-1AS* [20], *QPm.osu-1A* [49], *QPm.caas-1A* [50], and a meta-QTL *MQTL1* [29]. Additionally, on the same chromosome, during the same 2020–2021 season, we found another MTA at the 74.0 cM position that is co-located with five MTAs previously identified by Liu et al. [12], as well as SNP markers like tplb0041a22_935, Excalibur_c15098_591 [17], and BS00021714_51 [46].

Similarly, on chromosome 1B, an MTA was detected at 76.0 cM during 2022–2023 which shared the chromosomal region with previously identified loci *Qaprpm.cgb-1B* [51] and *QPm.osu-1B* [49], as well as five MTAs identified by Liu et al. [12]. 

Our analysis revealed that five SNPs detected between 132.0 and 144.0 cM on chromosome 2B during 2020–2021 and 2021–2022 were found in close proximity to previously detected QTLs *Qaprpm.cgb-2B* [51] and *QPm.crag-2B* [52]. 

On chromosome 3B, we detected twelve PM resistance-associated SNPs between 57.0 and 84.0 cM during 2021–2022 and 2022–2023. Comparison of the chromosome intervals with the previously identified loci such as *PM_3B1* and *PM_3B2* [16], *QPm.mgb-3BL.3* [20], *QPm.inra-3B* [67], *QPm.osu-3B* [49], *QPm.caas-3BS* [68], *CP2* [29], *QPm.mgb-3BL.3* [20], as well as other MTAs identified by Liu et al. [12] and Alemu et al. [46], showed that markers identified in our study possibly shared chromosomal intervals with *QPm.mgb-3BL.3* [20], as well as four MTAs identified by Liu et al. [12]. 

On chromosome 4A, an MTA was detected at 48.0 cM during 2021–2022. This MTA seems to share same chromosome region as previously identified QTL *QPm.mgb-4AL* [20] and a meta-QTL *MQTL11* [29]. 

The presence of two MTAs at 68.0 cM on chromosome 5B (detected during 2021–2022) may be associated with a previously reported broad-spectrum PM resistance gene *Pm16* [55], highlighting its potential for marker-assisted selection in breeding programs targeting PM resistance. 

Among the chromosomes examined, chromosome 6B stood out with the largest number of MTAs, totaling 57, one at 0.0 cM while remaining within the 59.0 to 65.0 cM region. The PM resistance MTA at 0.0 cM appears to overlap with a previously identified QTL on chromosome 6B from the Swedish winter wheat cultivar Folke [53]. However, no previously identified loci were found in second region, suggesting the presence of novel genes or regulatory elements associated with PM resistance in this region. 

On chromosome 7A, we identified six distinct MTAs at various positions (at 57.0, 64.0, 65.0, 73.0, 74.0, and 150.0 cM) during the 2020–2021 and 2022–2023 seasons. It appears that the MTAs located at 57.0, 64.0, 65.0, 73.0, and 74.0 are associated with a previously repetitively detected locus associated with QTLs *QPm.mgb-7AS* [20], *QPm.caas-7AS* [54] and a meta-QTL *MQTL21* [29]. Conversely, the MTA at 150.0 appears to be associated with QTLs *QPm.icg-7A* cM [17], *QPm.mgb-7AL* [20], and a meta-QTL *MQTL22* [29]. Simeone et al. [20] found that *QPm.mgb-7AL*, which appears associated with the second region detected on chromosome arm 7AL in this study, co-localizes with *Pm* resistance genes *Pm1* [56], *Pm37* [57], *Pm59* [58], and *Pm60* [59]. Notably, they also discovered a *Pm3-like* disease resistance protein gene in close proximity (within 10 kb) of this locus, suggesting that this locus holds significant promise as a target for marker-assisted selection. 

Chromosome 7B exhibited four MTAs, with one MTA detected at 73.0 cM during 2022–2023 and the other three detected at 136.0 cM during 2021–2022. A QTL *QPm.mgb-7BL* [20] has been previously reported in vicinity of the MTA detected at 73.0 cM position on chromosome 7B. Previously, Simeone et al. [20] revealed that *QPm.mgb-7BL* co-localizes with *Pm* resistance genes *PmE* [60], *Pm40* [61] and *Pm47* [62]. It appears that markers detected at 136.0 cM position represent a novel PM resistance locus since no resistance loci has been observed in this region previously.

In addition to the above-discussed MTAs, a number of other novel MTAs were detected on chromosomes 3A (at 158.0 cM, detected during 2022–2023), 4B (at 66.0 cM, detected during 2022–2023), and 5A (at 144.0 cM, detected during 2021–2022). No QTLs/MTAs or genes were previously reported by other studies in their vicinity. These findings emphasize the importance of further investigations focused on these and other novel MTA regions such as those on chromosome 6B (between 59.0 and 65.0 cM, detected during 2020–2021 and 2021–2022) for a deeper understanding of the genetic mechanisms underlying PM resistance.

The co-localization of MTAs with previously identified genetic markers, MTAs, QTLs and genes provides further evidence for their involvement in the resistance against PM. Identified novel MTAs could be valuable for diversifying the sources of genetic resistance in breeding programs and provide valuable targets to enhance PM resistance by combining with repetitively detected loci using the associated SNP markers identified in this study. Moreover, these findings provide valuable information for breeders to prioritize genomic regions and genetic markers associated with PM resistance and contribute to a better understanding of the genetic basis of PM resistance in common wheat.

### 3.3. Candidate Genes for PM Resistance

To identify potential target genes for breeding, GWAS is often used as a starting point [69]. However, a challenge arises when significant markers associated with the trait of interest encompass a wide range of genes within their confidence intervals, making it difficult to pinpoint the exact causal genes [36]. Thus, in this study, we addressed this issue by focusing on MTAs found associated with genomic intervals carrying disease resistance-associated candidate genes. A total of 30 out of the 96 candidate genes broadly represent twelve plant protein families that exhibited clear connections to disease resistance and host–pathogen interactions. These proteins have been previously reported to impart disease resistance to various pathogens such as *Blumeria graminis* f. sp. *tritici*, *Fusarium graminearum*, *Puccinia striiformis*, *Bipolaris sorokiniana*, *Puccinia triticina*, *Parastagonospora nodorum* and *Pyrenophora tritici*-*repentis* in plants [70,71,72,73,74,75,76,77,78,79]. 

It may be recalled that 30 candidate genes encode proteins with distinctive domains, including the ABC transporter-like domain, ATP-binding domain, ankyrin repeat, aspartic peptidase A1 family, peroxidase, cytochrome P450, disease resistance protein (NB-LRR), glutathione S-transferase, NB-ARC P-loop-containing nucleoside triphosphate hydrolase, kinase-like domain superfamily, wall-associated receptor kinase, galacturonan-binding domain, zinc finger proteins, and F-box superfamily domain (Table S2), which are widely recognized as essential components of disease resistance mechanisms. These results reinforce findings from previous research. For instance, Peng and Yang’s [80] comprehensive analysis delved into ABC, NLR, and START genes in hexaploid wheat, revealing their co-localization with disease resistance quantitative trait loci (QTLs) associated with leaf rust resistance. Molecular characterization of the leaf rust-resistance gene *Lr34*, which encodes an ABC transporter with transmembrane (TM) and nucleotide binding site (NB) domains, was elucidated in studies by Krattinger et al. [81,82]. Similarly, *Lr14a*, a race-specific leaf rust resistance gene, was found to encode an ANKTM protein [83]. The YrU1 protein, conferring wheat stripe rust resistance, also featured an integrated ANK domain from ANKTM proteins, suggesting their role in disease resistance [83,84]. The up-regulation of A1 aspartic peptidase and G1 families during *Zymoseptoria tritici* infection [72] underscores their defensive roles. Similarly, peroxidases’ positive contribution against *Puccinia striiformis* infection [85] highlights their significance in immunity. Disease sensitivity genes like *Tsn1* which encode a protein with S/TPK and NBS-LRR domains [71,74] reveal their significance in recognizing necrotrophic effectors. The critical role of the NB-ARC domain in regulating R protein activity [70] deepens our understanding of pathogen recognition. Wall-associated receptor kinases (WAKs), exemplified by *Stb6* [73] and *TaWAK6* [76], emerge as potent defenders against *Septoria tritici* blotch and leaf rust. During defense, identified proteins exhibit versatile functions [75,79], encompassing stress resilience, apoptosis, transcription, and interactions. The F-box family protein, known for its role in numerous biological processes, including biotic stress resistance [86], highlights the multifaceted engagement of these proteins in wheat’s resistance responses. However, despite the identification of several strong SNPs associated with PM resistance, no candidate genes were found in genomic intervals of 48 MTAs which may potentially carry other novel structural variants responsible for regulating plant defense against *Bgt* and wheat-*Bgt* interaction; however, further investigations are required to determine the significance and potential functional relevance of these specific SNPs in *Bgt* resistance.

In conclusion, our findings unveiled the intricate genetic landscape of PM resistance within the WAMI panel. This collection showcases a valuable pool of significant PM resistance MTAs/genes embedded in elite genetic backgrounds. Finally, through a comparison with previous studies on disease resistance in wheat, we have investigated the functions of key MTAs.

## 4. Materials and Methods

### 4.1. Plant Material

The WAMI panel, consisting of 286 genetically diverse and elite advanced wheat lines, was utilized for GWAS of PM resistance. The panel was assembled and distributed through the International Wheat Improvement Network (IWIN) by the International Maize and Wheat Improvement Center (CIMMYT) and possesses a narrow range of variation for days to heading and plant height which is appropriate for gene discovery without the confounding effects of phenology and plant height [63]. The seed of the WAMI panel was obtained from CIMMYT. A highly PM-susceptible common wheat cultivar WL711 [87] was used as a susceptible check and disease spreader. 

### 4.2. Experimental Design and Trait Evaluation

The field evaluations of genotypes were conducted at the research farm (30.75° N, 77.30° E) of Eternal University, Baru Sahib, Himachal Pradesh, India, spanning three consecutive growing seasons (2020–2021, 2021–2022, and 2022–2023). The Baru Sahib PM disease nursery research farm is situated in a north Indian hilly state, Himachal Pradesh, and is known for climatic conditions favorable for the natural infection, growth, and the development of PM [88]. During the PM disease development period, the temperature and relative humidity in Baru Sahib range between 15 and 22 °C (max. 30 °C) and 70 and 90%, respectively. The region is a natural hot spot for PM occurrence evidenced by data from the past 15 years (Dr. H. S. Dhaliwal, personal communication).

The experiments adhered to a randomized block design and were replicated twice. Random assignment of lines to each replication was carried out using the Fisher and Yates Random Table method [89]. Fertilizers included 120 kg N, 60 kg P_2_O_5_, and 40 kg K_2_O. Except N, fertilizers were thoroughly applied at the time of sowing. The application of nitrogen was divided into three doses: half at sowing, one-fourth at the first irrigation (21 days after sowing), and the remaining one-fourth at the second irrigation (45 days after sowing). 

A mixed population of the *Bgt* isolates was used in their natural state to create epiphytotic conditions in disease nurseries, which enhanced the severity of the disease. For this purpose, two rows of WL711, a highly PM-susceptible cultivar, were planted around the field, with an additional row seeded between the plots one month before sowing the WAMI panel in November each year. The WL711 was also sown alongside the experimental materials during main season sowing. Planting distances were maintained at 20 cm between rows and 5 cm between plants. 

Disease severity was scored using a rating scale of 1 to 9, following the method outlined by Bennett and Westcott [90], with some modifications on randomly chosen five plants from each plot. At this scale, level 1 denotes a severity of less than 1%, while level 2 corresponds to a range of 1–5%. The severity increases gradually through the scale, with level 9 indicating a severity of over 90%. Additionally, there’s a distinct note that severity scores falling within the range of 1 to 4 are denoted as resistance (Figure S1). The entire panel was rated once the powdery mildew reached its maximum expression, corresponding to a score of 9, observed on the susceptible check cultivar WL711. This approach enabled the tracking of disease progression over time and the identification of an appropriate growth stage exhibiting varying degrees of resistance or susceptibility to PM. 

### 4.3. DNA Extraction and Genotyping

DNA extraction, genotyping of samples and data processing were performed as described previously [91]. Briefly, DNA was extracted from fresh leaves of each line using the CTAB procedure, as outlined by Saghai-maroof et al. [92]. Subsequently, genotyping was conducted at the USDA-ARS Small Grain Genotyping Center, Fargo, utilizing the Illumina 90K Infinium iSelect assay (Illumina Inc., San Diego, CA, USA) [43]. The SNP calling process utilized the default clustering algorithm integrated into Genome Studio v2011.1 (Illumina Inc., San Diego, CA, USA), resulting in the identification of a total of 26,814 bi-allelic SNPs [91,93]. To uphold data quality standards, SNPs characterized by a minor allele frequency (MAF) lower than 0.05 were omitted from the analysis, alongside monomorphic and low-quality SNPs. This meticulous filtration procedure led to the retention of approximately 21,132 polymorphic SNPs [91,94], which were subsequently harnessed for the purpose of conducting the GWAS in this study.

### 4.4. Descriptive Statistics Analyses

Pearson’s correlation coefficients analysis was performed using the Agricolae (version 1.2–4) package of the R (version 4.0.3) software [95].Variance components were estimated using restricted maximum likelihood (REML) method implemented in META-R software v6.0.4 [96]. Covariance parameter estimates were used as unbiased genetic variance component estimates for calculating broad-sense heritability (*H*^2^) following Alemu et al. [46]. 

The BLUP values were obtained using the ‘lme4′ package [97] in R [95]. Descriptive statistics, such as the mean, standard deviation, and coefficient of variation (CV), were calculated using SPSS v. 17.0 (SPSS Inc., Chicago, IL, USA, 2008). 

### 4.5. Population Structure, Kinship Matrix and Principal Components Analyses

Population structure (population structure matrix or Q matrix) were modeled using principal components analysis (PCA) utilizing the genotypic data belonging to a total of 21,132 high-quality SNPs as described earlier [98]. The relatedness matrix (Kinship or K matrix) was calculated with R (version 4.0.3) software [95] employing the parameters suggested by VanRaden [99] and Yin et al. [100]. The optimal numbers of PCAs were determined using the Bayesian information criterion (BIC) [101]. The first two principal components were used to create a scatter plot that visualized the distribution of genotypes.

### 4.6. Genome-Wide Association Analyses

Genome-wide association analyses were conducted using a dataset of 21,132 high-quality SNPs available from the CIMMYT, Mexico website (https://data.cimmyt.org/dataset.xhtml?persistentId=hdl:11529/10714; accessed on 24 July 2023) [91]. Pairwise squared allele-frequency correlations (r^2^) between SNP markers were calculated using the TASSEL (Trait Analysis by Association, Evolution, and Linkage) software version 5.2.91 with a sliding window size of 100. To assess LD between loci, r^2^ values were plotted against genetic distance in cM. The LD decay curve was fitted using a smoothing spline regression line at the genome level, following the method outlined by Hill and Weir [45] and implemented in the R environment with a script previously employed by Marroni et al. [102].

Single- and multilocus models including the general linear model (GLM) [98], mixed linear model (MLM) [103], and the fixed and random model circulating probability unification (FarmCPU) [104] method were fitted to identify marker–trait associations (MTAs). Among these, FarmCPU was selected based on the best fit and used for the final GWAS. In FarmCPU, both fixed and random effects are incorporated to improve the accuracy of association mapping. It is a two-step procedure where the fixed effect model is initially fitted to control for population structure, and then the random effect model is used to further account for relatedness among individuals. The rMVP (R based Memory-efficient, Visualization-enhanced, and Parallel-accelerated Tool For Genome-Wide Association Study) software version 1.0.0 [100] was employed for the analysis.

A significance threshold of *p* < 0.001 (−log10(P) > 3.0) was applied to identify statistically significant MTAs as used previously [12,46,105,106]. 

### 4.7. Identification of Candidate Genes

To identify potential candidate genes, we focused on the most significant MTAs. The marker sequences associated with these MTAs were aligned with the wheat genome assembly IWGSC v.1 downloaded from the Ensembl database. Specifically, we analyzed a 200 kb window around each MTA marker (100 kb on each side) to extract highly significant and annotated candidate genes following Singh et al. [106] and Feng et al. [107]. A 200 kb window is a reasonable size to capture regulatory hubs that may be involved in the expression of a candidate genes. It is also a large enough window to capture multiple genes, which can be helpful for identifying co-expressed genes that may be involved in the same biological pathway. For the gene ontology (GO) annotation of these candidate genes, we utilized IWGSC (http://www.wheatgenome.org, accessed on 5 August 2023), which offered the requisite information for annotating the GO terms associated with the identified candidate genes.

## 5. Conclusions

Incorporating the insights gleaned from the three-year investigation, a focused set of significant MTAs emerged, each holding the potential to drive advancements in resistance to PM disease in wheat. Notably, recurrent novel MTAs, consistently observed on chromosome 6B across 2020–2021 and 2021–2022 (Table 3), underscore the enduring influence of key genomic regions on PM resistance. By harnessing the knowledge embedded within these MTAs, breeders can refine their selection processes, develop tailored markers, and ultimately expedite the development of superior PM-resistant cultivars through precision-guided approaches. This study not only sheds light on the genetic architecture underpinning PM resistance but also paves the way for innovative and accelerated wheat breeding programs that align with the demands of sustainable and resilient agricultural practices. Moreover, we identified potential candidate genes involved in disease resistance mechanisms in wheat by analyzing significant SNPs and their associated gene sequences.

## Figures and Tables

**Table 1:** The descriptive statistics and heritability for the PM disease severity in the wheat association mapping initiative (WAMI) panel containing 286 wheat accessions screened at Baru Sahib PM disease nurseries in Himachal Pradesh, India during three field seasons, spanning from 2020 to 2023.

Source	2020–2021	2021–2022	2022–2023
Min	1	2.5	1.5
Max	8.5	8.3	9
Mean	5.3	5.1	5.3
LSD	1.2	1.4	1.3
CV	8.4	12.2	9.0
Heritability	0.89	0.65	0.81

Min: minimum; Max: maximum; LSD: least significant difference; CV: coefficient of variation.

**Table 2:** Combined analysis of variance (ANOVA) table for PM disease severity in the wheat association mapping initiative (WAMI) panel containing 286 wheat accessions.

Source of Variation	Df	MS
Genotypes	285	4.9 **
Environments (years)	2	52.7 **
Replications	3	8.5
Genotype × Environment	570	1.22 **

Df: degrees of freedom; MS: mean squares; significant at ** *p* ≤ 0.001.

**Table 3:** Details of marker–trait associations (MTAs) detected for resistance against PM in the WAMI panel during 2020–2021, 2021–2022, and 2022–2023.

Marker	Chr	Pos (cM) ^1^	Pos (bp) ^2^	Effect	*p*-Value	MAF	Reference ^3^
**2020–2021**							
RAC875_c1710_376 ^$^	1A	27.0	9,103,721	−0.55	0.0004	0.28	[20,29,47,48,49,50]
Kukri_c35200_895 ^$^	1A	74.0	459,514,896	−0.59	0.0003	0.43	[12,17,46]
BS00080318_51 ^$^	2B	132.0	763,842,555	0.32	0.0003	0.43	[51,52]
Excalibur_c5438_274 ^$^	2B	142.0	774,958,099	0.30	0.0003	0.43	[51,52]
Excalibur_rep_c109577_698 ^$^	2B	144.0	775,368,259	−0.27	0.0008	0.16	[51,52]
RAC875_rep_c83950_222 ^$^	2B	144.0	775,053,184	−0.29	0.0003	0.42	[51,52]
Ra_c22493_190 ^$^	6B	0.0	6,196,794	−0.48	0.0006	0.42	[53]
Excalibur_rep_c106789_271	6B	59.0	153,957,925	−0.33	0.0004	0.17	PNM
RAC875_c25489_1208	6B	59.0	153,959,656	−0.31	0.0008	0.42	PNM
wsnp_Ex_c39304_46635517	6B	59.0	151,609,970	−0.34	0.0003	0.38	PNM
wsnp_Ex_rep_c102044_87296690	6B	59.0	153,959,656	−0.32	0.0005	0.42	PNM
wsnp_Ex_rep_c102044_87297599	6B	59.0	153,957,925	−0.34	0.0003	0.42	PNM
wsnp_Ra_c48999_54089942	6B	59.0	153,956,896	−0.31	0.0008	0.36	PNM
Excalibur_c3795_198 ^$^	6B	60.0	156,266,046	−0.31	0.0005	0.13	PNM
Ra_c14852_1487	6B	60.0	156,266,570	−0.32	0.0003	0.42	PNM
RAC875_c66770_208	7A	57.0	26,879,337	−0.58	0.0004	0.42	[20,29,54]
Excalibur_c7538_2718 ^$^	7A	65.0	35,602,223	−0.48	0.0004	0.10	[20,29,54]
wsnp_Ex_c35_77935 ^$^	7A	73.0	41,960,564	−0.36	0.0002	0.430	[20,29,54]
BS00023055_51	7A	74.0	41,959,666	−0.32	0.0004	0.28	[20,29,54]
**2021–2022**							
Excalibur_c7971_1573	2B	144.0	775,371,388	0.04	0.0009	0.14	[51,52]
BS00011728_51 ^$^	3B	57.0	59,064,340	−0.28	0.0000	0.10	[12,20]
BS00022741_51 ^$^	3B	61.0	66,953,509	−0.26	0.0009	0.16	[12,20]
Excalibur_c21372_142	3B	61.0	66,954,023	−0.26	0.0009	0.29	[12,20]
Tdurum_contig51993_52 ^$^	3B	61.0	66,953,509	−0.26	0.0009	0.20	[12,20]
BS00011869_51	3B	71.0	426,452,313	−0.23	0.0003	0.22	[12,20]
Excalibur_c80041_400 ^$^	3B	71.0	429,610,611	−0.26	0.0000	0.43	[12,20]
Kukri_c21818_519 ^$^	3B	71.0	556,059,780	−0.23	0.0002	0.36	[12,20]
RAC875_c58159_989	3B	71.0	569,425,136	−0.19	0.0008	0.43	[12,20]
wsnp_Ku_c21818_31604716 ^$^	3B	71.0	556,059,565	−0.24	0.0001	0.49	[12,20]
BobWhite_c27944_234 ^$^	4A	48.0	109,443,802	−0.23	0.0007	0.23	[20,29]
Ex_c17894_1159 ^$^	4A	48.0	654,418,148	−0.22	0.0005	0.42	[20,29]
Excalibur_rep_c66815_273	4A	48.0	104,375,678	−0.22	0.0005	0.44	[20,29]
GENE-2637_94 ^$^	4A	48.0	164,633,479	−0.23	0.0008	0.43	[20,29]
IAAV3906 ^$^	4A	48.0	113,854,919	−0.22	0.0006	0.43	[20,29]
IAAV8784	4A	48.0	135,358,615	−0.22	0.0005	0.43	[20,29]
IACX1896 ^$^	4A	48.0	109,431,746	−0.22	0.0007	0.35	[20,29]
Kukri_c44469_1240	4A	48.0	136,432,865	−0.22	0.0005	0.44	[20,29]
Kukri_c48155_158 ^$^	4A	48.0	120,605,031	−0.22	0.0005	0.43	[20,29]
RAC875_c22562_429 ^$^	4A	48.0	111,292,238	−0.23	0.0003	0.13	[20,29]
RAC875_rep_c74695_101 ^$^	4A	48.0	164,632,278	−0.23	0.0008	0.38	[20,29]
tplb0035b22_184	4A	48.0	135,614,180	−0.22	0.0005	0.43	[20,29]
wsnp_BE442869A_Ta_2_1	4A	48.0	140,134,106	−0.22	0.0005	0.42	[20,29]
wsnp_Ex_c10527_17198865 ^$^	4A	48.0	115,570,013	−0.22	0.0005	0.43	[20,29]
wsnp_Ex_c1387_2659020 ^$^	4A	48.0	115,912,754	−0.22	0.0005	0.42	[20,29]
wsnp_Ex_c14529_22547438	4A	48.0	115,913,618	−0.22	0.0005	0.44	[20,29]
wsnp_Ex_c1865_3515470 ^$^	4A	48.0	136,894,495	−0.22	0.0005	0.20	[20,29]
wsnp_Ex_c36141_44153175 ^$^	4A	48.0	111,091,261	−0.23	0.0007	0.44	[20,29]
wsnp_Ex_c4286_7734046	4A	48.0	114,744,375	−0.22	0.0006	0.43	[20,29]
wsnp_Ex_c43734_49968808 ^$^	4A	48.0	211,000,047	−0.24	0.0005	0.42	[20,29]
wsnp_Ex_rep_c69890_68851948 ^$^	4A	48.0	105,249,095	−0.24	0.0005	0.43	[20,29]
Tdurum_contig51134_191 ^$^	5A	144.0	698,501,228	−0.36	0.0000	0.42	PNM
RAC875_c19099_308 ^$^	5B	68.0	519,153,210	0.24	0.0007	0.42	[55]
Tdurum_contig53926_455 ^$^	5B	68.0	516,469,300	0.26	0.0006	0.38	[55]
BS00046963_51	6B	59.0	150,665,070	−0.19	0.0006	0.33	PNM
Excalibur_rep_c106789_271	6B	59.0	153,957,925	−0.20	0.0005	0.41	PNM
Kukri_c38732_225 ^$^	6B	59.0	151,131,387	−0.18	0.0007	0.42	PNM
wsnp_Ex_rep_c102044_87296690	6B	59.0	153,959,656	−0.19	0.0007	0.42	PNM
wsnp_Ex_rep_c102044_87297599	6B	59.0	153,957,925	−0.20	0.0004	0.43	PNM
Ra_c14852_1487 ^$^	6B	60.0	156,266,570	−0.18	0.0006	0.44	PNM
BS00040868_51 ^$^	6B	63.0	228,977,167	−0.24	0.0001	0.42	PNM
BobWhite_c34920_228	6B	64.0	249,498,169	−0.21	0.0006	0.42	PNM
BS00003955_51 ^$^	6B	64.0	260,548,319	−0.24	0.0001	0.33	PNM
BS00021686_51 ^$^	6B	64.0	174,566,651	−0.24	0.0003	0.30	PNM
BS00045761_51 ^$^	6B	64.0	227,070,547	−0.24	0.0002	0.33	PNM
BS00067871_51 ^$^	6B	64.0	261,585,651	−0.24	0.0002	0.41	PNM
BS00067873_51 ^$^	6B	64.0	261,585,682	−0.23	0.0003	0.17	PNM
BS00080544_51 ^$^	6B	64.0	234,560,063	−0.22	0.0007	0.43	PNM
Ex_c49055_617 ^$^	6B	64.0	260,548,488	−0.22	0.0005	0.44	PNM
Excalibur_c20503_382 ^$^	6B	64.0	257,568,556	−0.24	0.0001	0.40	PNM
Excalibur_c47738_334	6B	64.0	262,473,204	−0.22	0.0004	0.41	PNM
Excalibur_c5136_2314 ^$^	6B	64.0	231,677,537	−0.22	0.0003	0.45	PNM
Excalibur_c53834_416 ^$^	6B	64.0	257,993,802	−0.25	0.0000	0.40	PNM
Excalibur_c79066_165 ^$^	6B	64.0	232,168,465	−0.24	0.0002	0.43	PNM
GENE-2606_197 ^$^	6B	64.0	257,567,774	−0.25	0.0001	0.42	PNM
Kukri_c25377_106 ^$^	6B	64.0	174,567,929	−0.23	0.0005	0.15	PNM
Kukri_c38058_532	6B	64.0	257,567,774	−0.22	0.0009	0.41	PNM
Kukri_c52515_442	6B	64.0	259,877,872	−0.21	0.0005	0.43	PNM
Ra_c77985_260 ^$^	6B	64.0	214,089,373	−0.22	0.0008	0.46	PNM
RAC875_c12805_908 ^$^	6B	64.0	234,559,907	−0.23	0.0005	0.47	PNM
RAC875_c24962_1326	6B	64.0	229,280,173	−0.23	0.0003	0.39	PNM
RFL_Contig311_951	6B	64.0	231,354,317	−0.23	0.0003	0.40	PNM
TA005139-0719 ^$^	6B	64.0	234,559,694	−0.24	0.0004	0.40	PNM
wsnp_BQ161448B_Ta_2_1 ^$^	6B	64.0	261,530,574	−0.23	0.0002	0.39	PNM
wsnp_Ex_c1603_3056226	6B	64.0	221,813,724	−0.23	0.0004	0.41	PNM
wsnp_Ex_c23474_32717535 ^$^	6B	64.0	276,221,225	−0.25	0.0001	0.41	PNM
wsnp_Ex_c27934_37093614	6B	64.0	259,881,611	−0.24	0.0002	0.43	PNM
wsnp_Ex_c42372_48966781	6B	64.0	229,280,173	−0.21	0.0007	0.43	PNM
wsnp_Ex_c46160_51746546	6B	64.0	229,278,919	−0.23	0.0002	0.29	PNM
wsnp_Ex_rep_c103466_88415738	6B	64.0	274,209,042	−0.24	0.0002	0.41	PNM
wsnp_Ex_rep_c103466_88415994	6B	64.0	274,208,315	−0.22	0.0006	0.21	PNM
wsnp_Ex_rep_c103497_88437811 ^$^	6B	64.0	277,165,618	−0.24	0.0001	0.43	PNM
wsnp_Ex_rep_c68480_67305954	6B	64.0	259,292,578	−0.24	0.0001	0.40	PNM
wsnp_JD_c6448_7610859	6B	64.0	257,993,802	−0.24	0.0001	0.46	PNM
wsnp_Ku_c27423_37369145	6B	64.0	259,883,491	−0.21	0.0005	0.46	PNM
wsnp_Ra_c16850_25605248	6B	64.0	262,473,278	−0.23	0.0002	0.23	PNM
wsnp_Ra_c33358_42248399 ^$^	6B	64.0	257,993,727	−0.22	0.0004	0.40	PNM
wsnp_Ra_rep_c111161_93528347 ^$^	6B	64.0	277,163,269	−0.21	0.0005	0.17	PNM
wsnp_Ra_rep_c73725_71801179	6B	64.0	274,207,889	−0.21	0.0006	0.46	PNM
wsnp_Ra_rep_c73725_71801237	6B	64.0	274,207,831	−0.21	0.0005	0.31	PNM
wsnp_Ra_rep_c73731_71807419	6B	64.0	257,996,714	−0.24	0.0001	0.38	PNM
BobWhite_c32911_243 ^$^	6B	65.0	255,268,901	−0.22	0.0003	0.47	PNM
Kukri_c24148_254 ^$^	7B	136.0	705,270,901	−0.16	0.0007	0.16	PNM
TA005284-0990 ^$^	7B	136.0	704,271,881	−0.16	0.0006	0.31	PNM
wsnp_JD_c13673_13606066 ^$^	7B	136.0	704,271,648	−0.17	0.0004	0.32	PNM
**2022–2023**							
IACX3595 ^$^	1B	76.0	539,562,016	0.17	0.0009	0.16	[12,49,51]
BS00030652_51	3A	158.0	693,002,205	−0.25	0.0008	0.42	PNM
Excalibur_c8284_580 ^$^	3B	57.0	736,672,449	−0.20	0.0009	0.42	[12,20]
IAAV6566	3B	84.0	736,712,583	−0.17	0.0003	0.47	[12,20]
Tdurum_contig8365_433	3B	84.0	736,672,399	−0.17	0.0005	0.14	[12,20]
Kukri_c2526_1375 ^$^	4B	66.0	523,447,728	−0.28	0.0004	0.43	PNM
Excalibur_c25471_225	7A	64.0	34,539,197	0.17	0.0002	0.47	[20,29,54]
BS00094965_51 ^$^	7A	150.0	669,729,091	−0.30	0.0002	0.44	[17,20,29,56,57,58,59]
Kukri_c30836_582	7B	73.0	699,427,808	0.16	0.0002	0.10	[20,60,61,62]

^1^ = Marker position (genetic) on consensus map in cM; ^2^ = marker position (physical) on chromosome in bp; ^3^ = MTA reported previously or found associated with a known powdery mildew resistance gene or region; ^$^ = marker with candidate genes that encode a protein; PNM = potentially novel marker–trait association. Associated SNP markers (above threshold) that were common over the years are underlined.

## Data Availability

The single-nucleotide polymorphism (SNP) genotyping data of the wheat association mapping initiative (WAMI) germplasm panel of spring wheat, which are used for the present study, have been published previously by Sukumaran et al. [91], and are available publicly to download from the link: http://hdl.handle.net/11529/10714 (accessed on 5 March 2023). All other data generated or analyzed during this study are included in this published article.

## Supplementary Materials

The following supporting information can be downloaded at: https://www.mdpi.com/article/10.3390/plants12223864/s1, Supplementary File 1.xlsx. Table S1: Details of candidate genes identified in genomic intervals of marker–trait associations detected during experimental years 2020–2021, 2021–2022 and 2022–2023. Table S2: Details of defense and host–pathogen-associated proteins encoded by candidate genes identified in genomic intervals of marker–trait associations detected during this study. Supplementary File 2.docx. Figure S1. Disease severity assessment scale.

## References

[1] Grote U., Fasse A., Nguyen T.T., Erenstein O. (2021). Food Security and the Dynamics of Wheat and Maize Value Chains in Africa and Asia. Front. Sustain. Food Syst..

[2] Shahbandeh M. Total Wheat Consumption Worldwide 2022/23. https://www.statista.com/statistics/1094056/total-global-rice-consumption/.

[3] Alam M.A., Mandal M.S.N., Wang C., Wanquan J. (2013). Chromosomal Location and SSR Markers of a Powdery Mildew Resistance Gene in Common Wheat Line N0308. Afr. J. Microbiol Res..

[4] Mwale V.M., Chilembwe H.C., Uluko H.C. (2014). Wheat Powdery Mildew (*Blumeria graminis* f. sp. tritici): Damage Effects and Genetic Resistance Developed in Wheat (Triticum aestivum). J. Plant Sci..

[5] Te Beest D.E., Paveley N.D., Shaw M.W., van den Bosch F. (2008). Disease-Weather Relationships for Powdery Mildew and Yellow Rust on Winter Wheat. Phytopathology.

[6] Mehta A., Basandrai A.K., Banyal D.K., Basandrai D. (2018). Effect of Weather Parameters on Powdery Mildew Development of Wheat at Different Location in Himachal Pradesh. Indian Phytopathol..

[7] Hartl L., Weiss H., Stephan U., Zeller F.J., Jahoor A. (1995). Molecular Identification of Powdery Mildew Resistance Genes in Common Wheat (*Triticum aestivum* L.). Theor. Appl. Genet..

[8] Keller M., Keller B., Schachermayr G., Winzeler M., Schmid J.E., Stamp P., Messmer M.M. (1999). Quantitative Trait Loci for Resistance against Powdery Mildew in a Segregating Wheat × Spelt Population. Theor. Appl. Genet..

[9] Hysing S.-C., Merker A., Liljeroth E., Koebner R.M.D., Zeller F.J., Hsam S.L.K. (2007). Powdery Mildew Resistance in 155 Nordic Bread Wheat Cultivars and Landraces. Hereditas.

[10] Hua W., Liu Z., Zhu J., Xie C., Yang T., Zhou Y., Duan X., Sun Q., Liu Z. (2009). Identification and Genetic Mapping of *pm42*, a New Recessive Wheat Powdery Mildew Resistance Gene Derived from Wild Emmer (*Triticum turgidum* var. dicoccoides). Theor. Appl. Genet..

[11] Hao Y., Parks R., Cowger C., Chen Z., Wang Y., Bland D., Murphy J.P., Guedira M., Brown-Guedira G., Johnson J. (2015). Molecular Characterization of a New Powdery Mildew Resistance Gene *Pm54* in Soft Red Winter Wheat. Theor. Appl. Genet..

[12] Liu N., Bai G., Lin M., Xu X., Zheng W. (2017). Genome-wide Association Analysis of Powdery Mildew Resistance in U.S. Winter Wheat. Sci. Rep..

[13] Li G., Cowger C., Wang X., Carver B.F., Xu X. (2019). Characterization of *Pm65*, a New Powdery Mildew Resistance Gene on Chromosome 2AL of a Facultative Wheat Cultivar. Theor. Appl. Genet..

[14] Li G., Xu X., Tan C., Carver B.F., Bai G., Wang X., Bonman J.M., Wu Y., Hunger R., Cowger C. (2019). Identification of Powdery Mildew Resistance Loci in Wheat by Integrating Genome-Wide Association Study (GWAS) and Linkage Mapping. Crop J..

[15] Jia M., Xu H., Liu C., Mao R., Li H., Liu J., Du W., Wang W., Zhang X., Han R. (2020). Characterization of the Powdery Mildew Resistance Gene in the Elite Wheat Cultivar Jimai 23 and Its Application in Marker-Assisted Selection. Front. Genet..

[16] Kang Y., Barry K., Cao F., Zhou M. (2020). Genome-Wide Association Mapping for Adult Resistance to Powdery Mildew in Common Wheat. Mol. Biol. Rep..

[17] Leonova I.N. (2019). Genome-Wide Association Study of Powdery Mildew Resistance in Russian Spring Wheat (*T. aestivum* L.) Varieties. Russ. J. Genet..

[18] McIntosh R.A., Dubcovsky J., Rogers W.J., Xia X.C., Raupp W.J. (2020). Catalogue of Gene Symbols for Wheat: 2020 Supplement. Annual Wheat Newsletter.

[19] McIntosh R.A., Hart G.E., Devos K.M., Gale M.D., Rogers W.J. (1998). Catalogue of Gene Symbols for Wheat. https://wheat.pw.usda.gov/ggpages/wgc/98/.

[20] Simeone R., Piarulli L., Nigro D., Signorile M.A., Blanco E., Mangini G., Blanco A. (2020). Mapping Powdery Mildew (*Blumeria graminis* f. sp. tritici) Resistance in Wild and Cultivated Tetraploid Wheats. Int. J. Mol. Sci..

[21] Pang Y., Wu Y., Liu C., Li W., St. Amand P., Bernardo A., Wang D., Dong L., Yuan X., Zhang H. (2021). High-Resolution Genome-Wide Association Study and Genomic Prediction for Disease Resistance and Cold Tolerance in Wheat. Theor. Appl. Genet..

[22] Du X., Xu W., Peng C., Li C., Zhang Y., Hu L. (2021). Identification and Validation of a Novel Locus, *Qpm-3BL*, for Adult Plant Resistance to Powdery Mildew in Wheat Using Multilocus GWAS. BMC Plant Biol..

[23] He H., Liu R., Ma P., Du H., Zhang H., Wu Q., Yang L., Gong S., Liu T., Huo N. (2021). Characterization of *Pm68*, a New Powdery Mildew Resistance Gene on Chromosome 2BS of Greek Durum Wheat TRI 1796. Theor. Appl. Genet..

[24] Hinterberger V., Douchkov D., Lück S., Kale S., Mascher M., Stein N., Reif J.C., Schulthess A.W. (2022). Mining for New Sources of Resistance to Powdery Mildew in Genetic Resources of Winter Wheat. Front. Plant Sci..

[25] Jin P., Guo X., Guo M., Li R., Li Q., Cheng P., Wang B. (2022). Genome-Wide Association Mapping of Resistance to Powdery Mildew in Regional Trials of Wheat Mainly from China. Plant Dis..

[26] Spielmeyer W., McIntosh R.A., Kolmer J., Lagudah E.S. (2005). Powdery Mildew Resistance and *Lr34*/*Yr18* Genes for Durable Resistance to Leaf and Stripe Rust Cosegregate at a Locus on the Short Arm of Chromosome 7D of Wheat. Theor. Appl. Genet..

[27] Lillemo M., Asalf B., Singh R.P., Huerta-Espino J., Chen X.M., He Z.H., Bjørnstad Å. (2008). The Adult Plant Rust Resistance Loci *Lr34*/*Yr18* and *Lr46*/*Yr29* are Important Determinants of Partial Resistance to Powdery Mildew in Bread Wheat Line Saar. Theor. Appl. Genet..

[28] Herrera-Foessel S.A., Singh R.P., Lillemo M., Huerta-Espino J., Bhavani S., Singh S., Lan C., Calvo-Salazar V., Lagudah E.S. (2014). *Lr67*/*Yr46* Confers Adult Plant Resistance to Stem Rust and Powdery Mildew in Wheat. Theor. Appl. Genet..

[29] Marone D., Russo M.A., Laidò G., De Vita P., Papa R., Blanco A., Gadaleta A., Rubiales D., Mastrangelo A.M. (2013). Genetic Basis of Qualitative and Quantitative Resistance to Powdery Mildew in Wheat: From Consensus Regions to Candidate Genes. BMC Genom..

[30] Lee J.H., Graybosch R.A., Peterson C.J. (1995). Quality and Biochemical Effects of a IBL/IRS Wheat-Rye Translocation in Wheat. Theor. Appl. Genet..

[31] Zeng F.-S., Yang L.-J., Gong S.-J., Shi W.-q., Zhang X.-J., Wang H., Xiang L.-B., Xue M.-F., Yu D.-Z. (2014). Virulence and Diversity of *Blumeria graminis* f. sp. tritici Populations in China. J. Integr. Agric..

[32] Bapela T., Shimelis H., Terefe T., Bourras S., Sánchez-Martín J., Douchkov D., Desiderio F., Tsilo T.J. (2023). Breeding Wheat for Powdery Mildew Resistance: Genetic Resources and Methodologies—A Review. Agronomy.

[33] Dhariwal R., Randhawa H.S., Bilichak A., Laurie J.D. (2022). Mapping Quantitative Trait Loci in Wheat: Historic Perspective, Tools, and Methods for Analysis. Accelerated Breeding of Cereal Crops.

[34] Bartoli C., Roux F. (2017). Genome-Wide Association Studies in Plant Pathosystems: Toward an Ecological Genomics Approach. Front. Plant Sci..

[35] Vagndorf N., Nielsen N.H., Edriss V., Andersen J.R., Orabi J., Jørgensen L.N., Jahoor A. (2017). Genomewide Association Study Reveals Novel Quantitative Trait Loci Associated with Resistance Towards *Septoria tritici* Blotch in North European Winter Wheat. Plant Breed..

[36] Juliana P., Singh R.P., Poland J., Mondal S., Crossa J., Montesinos-López O.A., Dreisigacker S., Pérez-Rodríguez P., Huerta-Espino J., Crespo-Herrera L. (2018). Prospects and Challenges of Applied Genomic Selection—A New Paradigm in Breeding for Grain Yield in Bread Wheat. Plant Genome.

[37] Muqaddasi Q.H., Zhao Y., Rodemann B., Plieske J., Ganal M.W., Röder M.S. (2019). Genome-wide Association Mapping and Prediction of Adult Stage *Septoria tritici* Blotch Infection in European Winter Wheat via High-Density Marker Arrays. Plant Genome.

[38] Stadlmeier M., Jørgensen L.N., Corsi B., Cockram J., Hartl L., Mohler V. (2019). Genetic Dissection of Resistance to the Three Fungal Plant Pathogens *Blumeria graminis*, *Zymoseptoria tritici*, and *Pyrenophora tritici-repentis* Using a Multiparental Winter Wheat Population. G3 Genes Genomes Genet..

[39] Huang B.E., Verbyla K.L., Verbyla A.P., Raghavan C., Singh V.K., Gaur P., Leung H., Varshney R.K., Cavanagh C.R. (2015). MAGIC Populations in Crops: Current Status and Future Prospects. Theor. Appl. Genet..

[40] Buckler E.S.t., Thornsberry J.M. (2002). Plant Molecular Diversity and Applications to Genomics. Curr. Opin. Plant Biol..

[41] Zhu C., Gore M., Buckler E.S., Yu J. (2008). Status and Prospects of Association Mapping in Plants. Plant Genome.

[42] Nelson R., Wiesner-Hanks T., Wisser R., Balint-Kurti P. (2018). Navigating Complexity to Breed Disease-Resistant Crops. Nat. Rev. Genet..

[43] Wang S., Wong D., Forrest K., Allen A., Chao S., Huang B.E., Maccaferri M., Salvi S., Milner S.G., Cattivelli L. (2014). Characterization of Polyploid Wheat Genomic Diversity using a High-Density 90,000 Single Nucleotide Polymorphism Array. Plant Biotechnol. J..

[44] Bolser D., Staines D.M., Pritchard E., Kersey P. (2016). Ensembl Plants: Integrating Tools for Visualizing, Mining, and Analyzing Plant Genomics Data. Methods Mol. Biol..

[45] Hill W.G., Weir B.S. (1988). Variances and Covariances of Squared Linkage Disequilibria in Finite Populations. Theor. Popul. Biol..

[46] Alemu A., Brazauskas G., Gaikpa D.S., Henriksson T., Islamov B., Jørgensen L.N., Koppel M., Koppel R., Liatukas Ž., Svensson J.T. (2021). Genome-Wide Association Analysis and Genomic Prediction for Adult-Plant Resistance to *Septoria Tritici* Blotch and Powdery Mildew in Winter Wheat. Front. Genet..

[47] Zeller F.J., Lutz J., Stephan U. (1993). Chromosome Location of Genes for Resistance to Powdery Mildew in Common Wheat (*Triticum Aestivum* L.) 1. Mlk and other Alleles at the Pm3 Locus. Euphytica.

[48] Li G., Carver B.F., Cowger C., Bai G., Xu X. (2018). *Pm223899*, a New Recessive Powdery Mildew Resistance Gene Identified in Afghanistan Landrace PI 223899. Theor. Appl. Genet..

[49] Chen Y., Hunger R.M., Carver B.F., Zhang H., Yan L. (2009). Genetic Characterization of Powdery Mildew Resistance in U.S. Hard Winter Wheat. Mol. Breed..

[50] Liang S.S., Suenaga K., He Z.H., Wang Z.L., Liu H.Y., Wang D.S., Singh R.P., Sourdille P., Xia X.C. (2006). Quantitative Trait Loci Mapping for Adult-Plant Resistance to Powdery Mildew in Bread Wheat. Phytopathology.

[51] Huang Q., Jing R., Wu X. (2008). QTL Mapping for Adult-Plant Resistance to Powdery Mildew in Common Wheat. Sci. Agric. Sin..

[52] Mingeot D., Chantret N., Baret P.V., Dekeyser A., Boukhatem N., Sourdille P., Doussinault G., Jacquemin J.M. (2002). Mapping QTL Involved in Adult Plant Resistance to Powdery Mildew in the Winter Wheat Line RE714 in Two Susceptible Genetic Backgrounds. Plant Breed..

[53] Lillemo M., Bjørnstad Å., Skinnes H. (2012). Molecular Mapping of Partial Resistance to Powdery Mildew in Winter Wheat Cultivar Folke. Euphytica.

[54] Lan C., Liang S., Wang Z., Yan J., Zhang Y., Xia X., He Z. (2009). Quantitative Trait Loci Mapping for Adult-Plant Resistance to Powdery Mildew in Chinese Wheat Cultivar Bainong 64. Phytopathology.

[55] Chen X.M., Luo Y.H., Xia X.C., Xia L.Q., Chen X., Ren Z.L., He Z.H., Jia J.Z. (2005). Chromosomal Location of Powdery Mildew Resistance Gene *Pm16* in Wheat using SSR Marker Analysis. Plant Breed..

[56] Singrün C., Hsam S.L., Hartl L., Zeller F.J., Mohler V. (2003). Powdery Mildew Resistance Gene *Pm22* in Cultivar Virest is a Member of the Complex *Pm1* Locus in Common Wheat (*Triticum aestivum* L. em Thell.). Theor. Appl. Genet..

[57] Perugini L.D., Murphy J.P., Marshall D., Brown-Guedira G. (2008). *Pm37*, a New Broadly Effective Powdery Mildew Resistance Gene from *Triticum timopheevii*. Theor. Appl. Genet..

[58] Tan C., Li G., Cowger C., Carver B.F., Xu X. (2018). Characterization of *Pm59*, a Novel Powdery Mildew Resistance Gene in Afghanistan Wheat Landrace PI 181356. Theor. Appl. Genet..

[59] Zou S., Wang H., Li Y., Kong Z., Tang D. (2018). The NB-LRR Gene *Pm60* Confers Powdery Mildew Resistance in Wheat. New Phytol..

[60] Ma Q., Luo P., Ren Z., Jiang H., Yang Z. (2010). Genetic Analysis and Chromosomal Location of Two New Genes for Resistance to Powdery Mildew in Wheat (*Triticum aestivum* L.). Acta Agron. Sin..

[61] Zhong S., Ma L., Fatima S.A., Yang J., Chen W., Liu T., Hu Y., Li Q., Guo J., Zhang M. (2016). Collinearity Analysis and High-Density Genetic Mapping of the Wheat Powdery Mildew Resistance Gene *Pm40* in PI 672538. PLoS ONE.

[62] Xiao M., Song F., Jiao J., Wang X., Xu H., Li H. (2013). Identification of the Gene *Pm47* on Chromosome 7BS Conferring Resistance to Powdery Mildew in the Chinese Wheat Landrace Hongyanglazi. Theor. Appl. Genet..

[63] Lopes M.S., Dreisigacker S., Peña R.J., Sukumaran S., Reynolds M.P. (2015). Genetic Characterization of the Wheat Association Mapping Initiative (WAMI) Panel for Dissection of Complex Traits in Spring Wheat. Theor. Appl. Genet..

[64] Singh S., Sehgal D., Kumar S., Arif M.A.R., Vikram P., Sansaloni C.P., Fuentes-Dávila G., Ortiz C. (2020). GWAS Revealed a Novel Resistance Locus on Chromosome 4D for the Quarantine Disease Karnal Bunt in Diverse Wheat Pre-Breeding Germplasm. Sci. Rep..

[65] Singh S., Mishra V.K., Kharwar R.N., Budhlakoti N., Ahirwar R.N., Mishra D.C., Kumar S., Chand R., Kumar U., Kumar S. (2020). Genetic Characterization for Lesion Mimic and other Traits in Relation to Spot Blotch Resistance in Spring Wheat. PLoS ONE.

[66] D’hoop B.B., Paulo M.J., Kowitwanich K., Sengers M., Visser R.G.F., van Eck H.J., van Eeuwijk F.A. (2010). Population Structure and Linkage Disequilibrium Unravelled in Tetraploid Potato. Theor. Appl. Genet..

[67] Bougot Y., Lemoine J., Pavoine M.T., Guyomar’ch H., Gautier V., Muranty H., Barloy D. (2006). A Major QTL Effect Controlling Resistance to Powdery Mildew in Winter Wheat at the Adult Plant Stage. Plant Breed..

[68] Jia A., Ren Y., Gao F., Yin G., Liu J., Guo L., Zheng J., He Z., Xia X. (2018). Mapping and Validation of a New QTL for Adult-Plant Resistance to Powdery Mildew in Chinese Elite Bread Wheat Line Zhou8425B. Theor. Appl. Genet..

[69] Dellaporta S.L., Wood J., Hicks J.B. (1983). Isolation of DNA from Higher Plants. Plant Mol. Biol. Rep..

[70] van Ooijen G., Mayr G., Kasiem M.M., Albrecht M., Cornelissen B.J., Takken F.L. (2008). Structure-Function Analysis of the NB-ARC Domain of Plant Disease Resistance Proteins. J. Exp. Bot..

[71] Faris J.D., Zhang Z., Lu H., Lu S., Reddy L., Cloutier S., Fellers J.P., Meinhardt S.W., Rasmussen J.B., Xu S.S. (2010). A Unique Wheat Disease Resistance-Like Gene Governs Effector-Triggered Susceptibility to Necrotrophic Pathogens. Proc. Natl. Acad. Sci. USA.

[72] Krishnan P., Ma X., McDonald B.A., Brunner P.C. (2018). Widespread Signatures of Selection for Secreted Peptidases in a Fungal Plant Pathogen. BMC Evol. Biol..

[73] Saintenac C., Lee W.-S., Cambon F., Rudd J.J., King R.C., Marande W., Powers S.J., Bergès H., Phillips A.L., Uauy C. (2018). Wheat Receptor-Kinase-Like Protein Stb6 Controls Gene-for-Gene Resistance to Fungal Pathogen *Zymoseptoria tritici*. Nat. Genet..

[74] Gupta P.K., Chand R., Vasistha N.K., Pandey S.P., Kumar U., Mishra V.K., Joshi A.K. (2018). Spot Blotch Disease of Wheat: The Current Status of Research on Genetics and Breeding. Plant Pathol..

[75] Noman A., Aqeel M., Khalid N., Islam W., Sanaullah T., Anwar M., Khan S., Ye W., Lou Y. (2019). Zinc Finger Protein Transcription Factors: Integrated Line of Action for Plant Antimicrobial Activity. Microb. Pathog..

[76] Dmochowska-Boguta M., Kloc Y., Zielezinski A., Werecki P., Nadolska-Orczyk A., Karlowski W.M., Orczyk W. (2020). *TaWAK6* Encoding Wall-Associated Kinase is Involved in Wheat Resistance to Leaf Rust Similar to Adult Plant Resistance. PLoS ONE.

[77] Yates S., Mikaberidze A., Krattinger S.G., Abrouk M., Hund A., Yu K., Studer B., Fouche S., Meile L., Pereira D. (2019). Precision Phenotyping Reveals Novel Loci for Quantitative Resistance to *Septoria Tritici* Blotch. Plant Phenomics.

[78] Mahmoudi Z., Taliei F., Ahangar L., Kheyrgoo M. (2021). Assessment of Salicylic Acid-Induced Resistance against *Septoria tritici* Blotch Disease on Wheat Using Real-Time PCR. J. Crop Prot..

[79] Lin P.-C., Pomeranz M.C., Jikumaru Y., Kang S.G., Hah C., Fujioka S., Kamiya Y., Jang J.-C. (2011). The Arabidopsis Tandem Zinc Finger Protein Attzf1 affects ABA- and GA-Mediated Growth, Stress and Gene Expression Responses. Plant J..

[80] Peng F.Y., Yang R.C. (2017). Prediction and Analysis of Three Gene Families Related to Leaf Rust (*Puccinia triticina*) Resistance in Wheat (*Triticum aestivum* L.). BMC Plant Biol.

[81] Krattinger S.G., Lagudah E.S., Spielmeyer W., Singh R.P., Huerta-Espino J., McFadden H., Bossolini E., Selter L.L., Keller B. (2009). A Putative ABC Transporter Confers Durable Resistance to Multiple Fungal Pathogens in Wheat. Science.

[82] Krattinger S.G., Lagudah E.S., Wicker T., Risk J.M., Ashton A.R., Selter L.L., Matsumoto T., Keller B. (2011). *Lr34* Multi-Pathogen Resistance ABC Transporter: Molecular Analysis of Homoeologous and Orthologous Genes in Hexaploid Wheat and Other Grass Species. Plant J. Cell Mol. Biol..

[83] Kolodziej M.C., Singla J., Sánchez-Martín J., Zbinden H., Šimková H., Karafiátová M., Doležel J., Gronnier J., Poretti M., Glauser G. (2021). A Membrane-Bound Ankyrin Repeat Protein Confers Race-Specific Leaf Rust Disease Resistance in Wheat. Nat. Commun..

[84] Wang H., Zou S., Li Y., Lin F., Tang D. (2020). An Ankyrin-Repeat and WRKY-Domain-Containing Immune Receptor Confers Stripe Rust Resistance in Wheat. Nat. Commun..

[85] Yang Y., Yu Y., Bi C., Kang Z. (2016). Quantitative Proteomics Reveals the Defense Response of Wheat against *Puccinia striiformis* f. sp. tritici. Sci. Rep..

[86] Kim H.S., Delaney T.P. (2002). Arabidopsis SON1 is an F-Box Protein that Regulates a Novel Induced Defense Response Independent of Both Salicylic Acid and Systemic Acquired Resistance. Plant Cell.

[87] Sharma M., Kaur S., Saluja M., Chhuneja P. (2016). Mapping and Characterization of Powdery Mildew Resistance Gene in Synthetic Wheat. Czech J. Genet. Plant Breed..

[88] Gautam A.K. (2015). Studies on Some Powdery Mildew of Himachal Pradesh, India. Australas. Mycol..

[89] Panse V.G., Sukhatme P.V. (1985). Statistical Methods for Agricultural Workers.

[90] Bennett F.G.A., Westcott B. (1982). Field Assessment of Resistance to Powdery Mildew in Mature Wheat Plants. Plant Pathol..

[91] Sukumaran S., Crossa J., Jarquín D., Lopes M., Reynolds M.P. (2016). Genomic and Pedigree Prediction with Genotype × Environment Interaction in Spring Wheat Grown in South and Western Asia, North Africa, and Mexico. G3-Genes Genomes Genet..

[92] Saghai-Maroof M.A., Soliman K.M., Jorgensen R.A., Allard R.W. (1984). Ribosomal DNA Spacer-Length Polymorphisms in Barley: Mendelian Inheritance, Chromosomal Location, and Population Dynamics. Proc. Natl. Acad. Sci. USA.

[93] Sukumaran S., Lopes M., Dreisigacker S., Reynolds M. (2018). Genetic Analysis of Multi-Environmental Spring Wheat Trials Identifies Genomic Regions for Locus-Specific Trade-Offs for Grain Weight and Grain Number. Theor. Appl. Genet..

[94] Ahirwar R.N., Mishra V.K., Chand R., Budhlakoti N., Mishra D.C., Kumar S., Singh S., Joshi A.K. (2018). Genome-Wide Association Mapping of Spot Blotch Resistance in Wheat Association Mapping Initiative (WAMI) Panel of Spring Wheat (*Triticum aestivum* L.). PLoS ONE.

[95] R Core Team (2013). R: A Language and Environment for Statistical Computing.

[96] Alvarado G., Rodríguez F.M., Pacheco A., Burgueño J., Crossa J., Vargas M., Pérez-Rodríguez P., Lopez-Cruz M.A. (2020). META-R: A Software to Analyze Data from Multi-Environment Plant Breeding Trials. Crop J..

[97] Bates D., Mächler M., Bolker B., Walker S. (2015). Fitting Linear Mixed-Effects Models using lme4. J. Stat. Softw..

[98] Price A.L., Patterson N.J., Plenge R.M., Weinblatt M.E., Shadick N.A., Reich D. (2006). Principal Components Analysis Corrects for Stratification in Genome-Wide Association Studies. Nat. Genet..

[99] VanRaden P.M. (2008). Efficient Methods to Compute Genomic Predictions. J Dairy Sci.

[100] Yin L., Zhang H., Tang Z., Xu J., Yin D., Zhang Z., Yuan X., Zhu M., Zhao S., Li X. (2021). rMVP: A Memory-Efficient, Visualization-Enhanced, and Parallel-Accelerated Tool for Genome-Wide Association Study. Genom. Proteom. Bioinform..

[101] Schwarz G. (1978). Estimating the Dimension of a Model. Ann. Stat..

[102] Marroni F., Pinosio S., Zaina G., Fogolari F., Felice N., Cattonaro F., Morgante M. (2011). Nucleotide Diversity and Linkage Disequilibrium in *Populus nigra cinnamyl alcohol dehydrogenase* (*CAD4*) Gene. Tree Genet. Genomes.

[103] Yu J., Pressoir G., Briggs W.H., Vroh Bi I., Yamasaki M., Doebley J.F., McMullen M.D., Gaut B.S., Nielsen D.M., Holland J.B. (2006). A Unified Mixed-Model Method for Association Mapping that Accounts for Multiple Levels of Relatedness. Nat. Genet..

[104] Liu X., Huang M., Fan B., Buckler E.S., Zhang Z. (2016). Iterative Usage of Fixed and Random Effect Models for Powerful and Efficient Genome-Wide Association Studies. PLoS Genet..

[105] Maccaferri M., El-Feki W., Nazemi G., Salvi S., Canè M.A., Colalongo M.C., Stefanelli S., Tuberosa R. (2016). Prioritizing Quantitative Trait Loci for Root System Architecture in Tetraploid Wheat. J. Exp. Bot..

[106] Singh S., Gaurav S.S., Vasistha N.K., Kumar U., Joshi A.K., Mishra V.K., Chand R., Gupta P.K. (2023). Genetics of Spot Blotch Resistance in Bread Wheat (*Triticum aestivum* L.) using five models for GWAS. Front. Plant Sci..

[107] Feng Y., Lu Q., Zhai R., Zhang M., Xu Q., Yang Y., Wang S., Yuan X., Yu H., Wang Y. (2016). Genome Wide Association Mapping for Grain Shape Traits in Indica Rice. Planta.

