# Supplementary material for: Genome-Wide Association Study Reveals Novel Powdery Mildew Resistance Loci in Bread Wheat
Source: Plants (Basel). 2023 Nov 15;12(22):3864. doi: 10.3390/plants12223864 (PMC10675159; doi:10.3390/plants12223864)
Supplement: Supplementary file 1 [file plants-12-03864-s001.zip › Supplementary File 2.pdf]

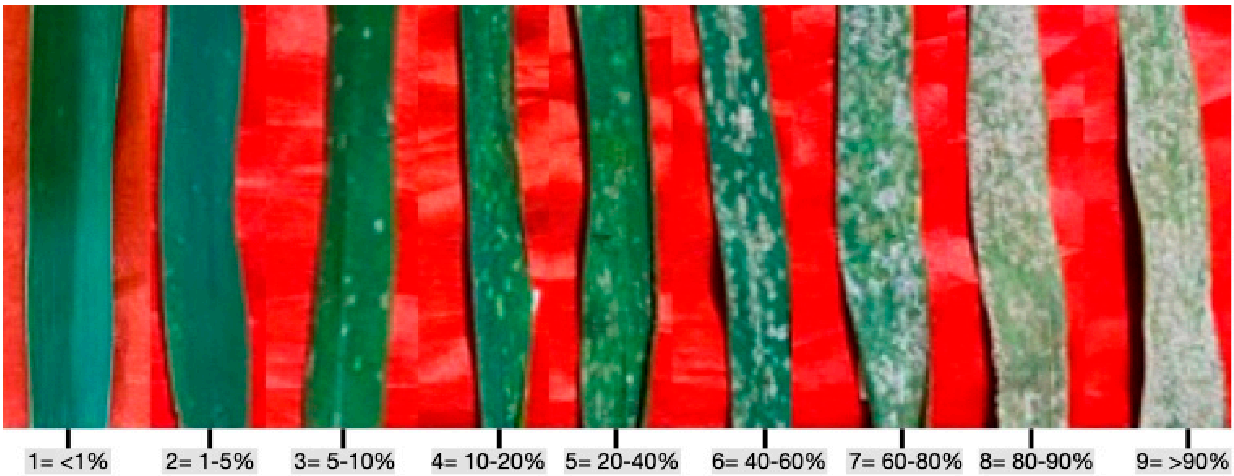

Figure S1. Disease severity assessment scale.

Disease severity assessment:

Disease severity was assessed using a rating scale ranging from 1 to 9 (Figure S1), following the method outlined by Bennett and Westcott [1], with some modifications. This scale encompasses level 1, indicating less than 1% severity, to level 9, denoting a severity exceeding 90%. Intermediate levels on the scale correspond to increasing disease severity.

Reference

Bennett, F.G.A.; Westcott, B. Field assessment of resistance to powdery mildew in mature wheat plants. *Plant Pathology* **1982**, *31*, 261-268, doi:https://doi.org/10.1111/j.1365-3059.1982.tb01277.x.
